# 2-[1-(4-Bromo­phen­yl)-2-nitro­eth­yl]hexa­noic acid

**DOI:** 10.1107/S1600536814006941

**Published:** 2014-04-05

**Authors:** Yanpeng Zhang, Can Zhang, Ai-Bao Xia

**Affiliations:** a State Key Laboratory Breeding Base of Green Chemistry-Synthesis Technology, Zhejiang University of Technology, Hangzhou 310014, People’s Republic of China

## Abstract

In the crystal structure of the title compoud, C_14_H_18_BrNO_4_, mol­ecules are linked by a strong O—H⋯O hydrogen bond and weaker C—H⋯O inter­actions. The benzene ring makes dihedral angles of 3.67 (3) and 72.63 (3)° with the carb­oxy­lic acid group and the nitro group, respectively.

## Related literature   

For related compounds, see: Wu *et al.* (2011 ▶); Nayak *et al.* (2013 ▶); Zhang *et al.* (2013 ▶); Thirunavukkarasu *et al.* (2014 ▶). For the asymmetric Michael reaction, which allows for the formation of two asymmetric centres, see: Enders *et al.* (2002 ▶); Hayashi *et al.* (2005 ▶); Keller *et al.* (2013 ▶).
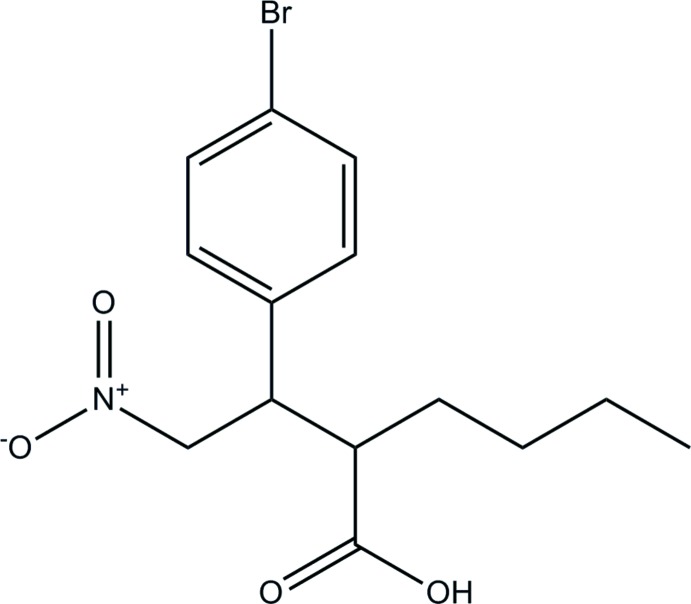


## Experimental   

### 

#### Crystal data  

C_14_H_18_BrNO_4_
*M* *_r_* = 344.20
Triclinic, 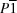

*a* = 7.2825 (11) Å
*b* = 8.7850 (13) Å
*c* = 13.026 (2) Å
α = 107.882 (3)°
β = 93.156 (3)°
γ = 101.555 (3)°
*V* = 770.9 (2) Å^3^
*Z* = 2
Mo *K*α radiation
μ = 2.68 mm^−1^
*T* = 140 K
0.25 × 0.20 × 0.15 mm

#### Data collection  

Bruker APEXII CCD diffractometer
Absorption correction: multi-scan (*SADABS*; Bruker, 2004 ▶) *T*_min_ = 0.510, *T*_max_ = 0.746
7818 measured reflections
4717 independent reflections
3078 reflections with *I* > 2σ(*I*)
*R*_int_ = 0.026

#### Refinement  

*R*[*F* ^2^ > 2σ(*F* ^2^)] = 0.045
*wR*(*F* ^2^) = 0.113
*S* = 0.99
4717 reflections
182 parameters
H-atom parameters constrained
Δρ_max_ = 0.77 e Å^−3^
Δρ_min_ = −0.57 e Å^−3^

### 

Data collection: *APEX2* (Bruker, 2004 ▶); cell refinement: *SAINT* (Bruker, 2004 ▶); data reduction: *SAINT*; program(s) used to solve structure: *SHELXS97* (Sheldrick, 2008 ▶); program(s) used to refine structure: *SHELXL2013* (Sheldrick, 2008 ▶); molecular graphics: *SHELXTL* (Sheldrick, 2008 ▶); software used to prepare material for publication: *SHELXTL*.

## Supplementary Material

Crystal structure: contains datablock(s) General, I. DOI: 10.1107/S1600536814006941/fj2666sup1.cif

Structure factors: contains datablock(s) I. DOI: 10.1107/S1600536814006941/fj2666Isup2.hkl

Supporting information file. DOI: 10.1107/S1600536814006941/fj2666Isup3.cml

CCDC reference: 994185

Additional supporting information:  crystallographic information; 3D view; checkCIF report

## Figures and Tables

**Table 1:** Hydrogen-bond geometry (Å, °)

*D*—H⋯*A*	*D*—H	H⋯*A*	*D*⋯*A*	*D*—H⋯*A*
C1—H1*B*⋯O1^i^	0.99	2.43	3.410 (3)	172
C1—H1*A*⋯O2^ii^	0.99	2.51	3.265 (3)	133
C3—H3⋯O3^iii^	1.00	2.69	3.679 (3)	169
C8—H8*B*⋯O2^iv^	0.98	2.60	3.501 (4)	153
O3—H3*A*⋯O4^v^	0.84	1.79	2.619 (2)	171

Symmetry codes: (i) 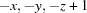; (ii) 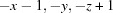; (iii) 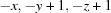; (iv) 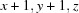; (v) 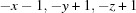.

**Fig. 1.**
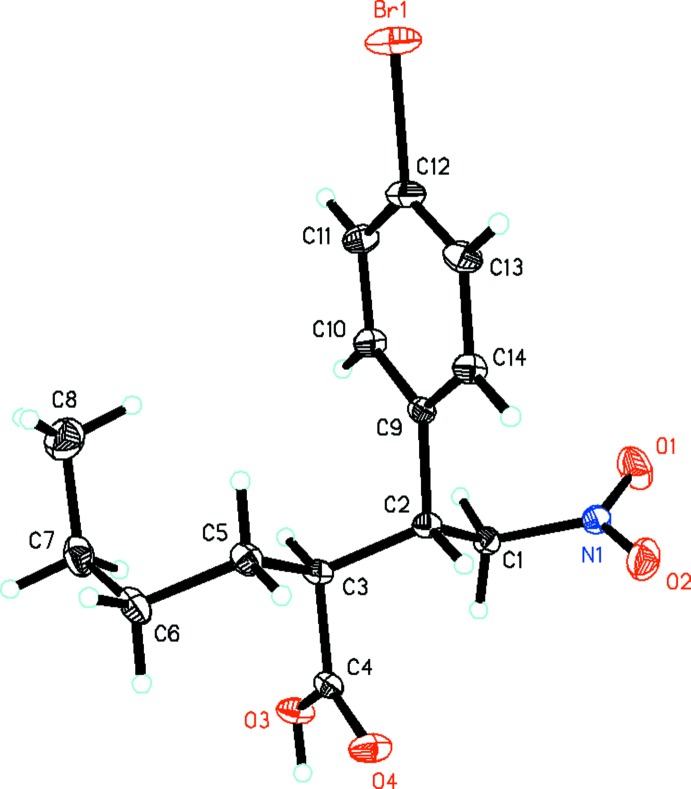
The asymmetric unit of the structure of the title compound, with the atomic labeling scheme. Displacement ellipsoids are drawn at the 50% probability level.

**Fig. 2.**
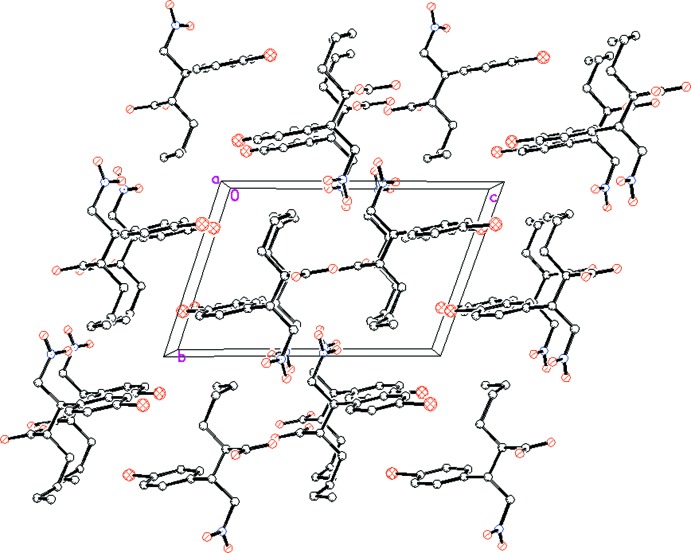
Unit cell packing of the title compound.

## supplementary crystallographic information

### 1. Comment

Michael addition can represent the initiating step of many complex inter- and intramolecular tandem processes. The use of the highly reactive nitroalkene as Michael acceptors opens the way to synthetically very useful C—C and C—*X* bond-forming reactions and subsequent transformations as is demonstrated by various applications(Enders *et al.*,2002). The title compound was obtained from the Michael addition of hexanal to (*E*)-1-bromo-4-(2-nitrovinyl)benzene in our laboratory. The crystal structure of the title compoud has been presented in this paper in Fig. 1. The C2···C3 distance being 1.545 (2) Å. The C1—C2—C3—C4 torsion angle of 59.43 (3)°. The C12···Br distance being 1.894 (2) Å. The C1···N1 distance being 1.492 (2) Å. The H3A—O3—C4 angle of 109.49 (3)°. In the crystal, molecules are linked by weak intermolecular O—H···O interactions. In addition,molecules are also linked by weak H3A—O4, H3A—C4 and H3A– H3A interactions respectively. The dihedral angle between the carboxylic acid group and the benzene ring is 3.67 (3)° and the dihedral angle between the nitro group and the benzene ring is 72.63 (3)°.

### 2. Experimental

*N*,*N*-Dimethylformamide(1.25 ml) was added to the mixture of hexanal (2.5 mmol) with (*E*)-1-bromo-4-(2-nitrovinyl)benzene(0.5 mmol) in the presence of D,*L*-proline (0.15 mmol) at room temperature with vigorous stirring. After 1 day, the mixture was extracted with DCM. Solvents were removed under vacuum and the residue was purified by column chromatography on silica gel(eluent:petroleum ether-ether). Then the addition product was oxidized into acid by H_2_O_2_. Suitable crystals were obtained by slow evaporation of a dichloromethane solution.

### 3. Refinement

H atoms were placed in calculated position with C—H ranging from 0.93 Å to 0.98 Å and refined using riding model with *U*_iso_(H)=1.2*U*_eq_ of the carrier atoms.

### Crystal data

**Table:**

C_14_H_18_BrNO_4_	*Z* = 2
*M**_r_* = 344.20	*F*(000) = 352
Triclinic, *P*1	*D*_x_ = 1.483 Mg m^−^^3^
*a* = 7.2825 (11) Å	Mo *K*α radiation, λ = 0.71073 Å
*b* = 8.7850 (13) Å	Cell parameters from 1805 reflections
*c* = 13.026 (2) Å	θ = 2.5–27.0°
α = 107.882 (3)°	µ = 2.68 mm^−^^1^
β = 93.156 (3)°	*T* = 140 K
γ = 101.555 (3)°	Block, colourless
*V* = 770.9 (2) Å^3^	0.25 × 0.20 × 0.15 mm

### Data collection

**Table:**

Bruker APEXII CCD diffractometer	3078 reflections with *I* > 2σ(*I*)
φ and ω scans	*R*_int_ = 0.026
Absorption correction: multi-scan (*SADABS*; Bruker, 2004)	θ_max_ = 30.7°, θ_min_ = 1.7°
*T*_min_ = 0.510, *T*_max_ = 0.746	*h* = −7→10
7818 measured reflections	*k* = −12→11
4717 independent reflections	*l* = −18→18

### Refinement

**Table:**

Refinement on *F*^2^	0 restraints
Least-squares matrix: full	Hydrogen site location: inferred from neighbouring sites
*R*[*F*^2^ > 2σ(*F*^2^)] = 0.045	H-atom parameters constrained
*wR*(*F*^2^) = 0.113	*w* = 1/[σ^2^(*F*_o_^2^) + (0.0579*P*)^2^] where *P* = (*F*_o_^2^ + 2*F*_c_^2^)/3
*S* = 0.99	(Δ/σ)_max_ = 0.005
4717 reflections	Δρ_max_ = 0.77 e Å^−^^3^
182 parameters	Δρ_min_ = −0.57 e Å^−^^3^

### Special details

**Table:**

Geometry. All e.s.d.'s (except the e.s.d. in the dihedral angle between two l.s. planes) are estimated using the full covariance matrix. The cell e.s.d.'s are taken into account individually in the estimation of e.s.d.'s in distances, angles and torsion angles; correlations between e.s.d.'s in cell parameters are only used when they are defined by crystal symmetry. An approximate (isotropic) treatment of cell e.s.d.'s is used for estimating e.s.d.'s involving l.s. planes.

### Fractional atomic coordinates and isotropic or equivalent isotropic displacement parameters (Å^2^)

**Table:**

	*x*	*y*	*z*	*U*_iso_*/*U*_eq_	
Br1	0.43884 (4)	0.25204 (4)	1.00399 (2)	0.04824 (13)	
N1	−0.2878 (3)	−0.0037 (2)	0.57284 (16)	0.0281 (4)	
O1	−0.2159 (3)	−0.1173 (2)	0.53116 (18)	0.0503 (5)	
O2	−0.4281 (3)	−0.0168 (2)	0.61913 (17)	0.0433 (5)	
O3	−0.2581 (2)	0.5058 (2)	0.50126 (13)	0.0309 (4)	
H3A	−0.3560	0.5143	0.4683	0.046*	
O4	−0.4590 (2)	0.4623 (2)	0.61743 (15)	0.0363 (4)	
C1	−0.2028 (3)	0.1613 (3)	0.56541 (18)	0.0239 (5)	
H1A	−0.2850	0.1860	0.5125	0.029*	
H1B	−0.0781	0.1604	0.5389	0.029*	
C2	−0.1790 (3)	0.2947 (3)	0.67608 (17)	0.0211 (4)	
H2	−0.3015	0.2804	0.7070	0.025*	
C3	−0.1361 (3)	0.4661 (3)	0.66194 (17)	0.0222 (4)	
H3	−0.0170	0.4809	0.6278	0.027*	
C4	−0.2969 (3)	0.4783 (3)	0.58874 (18)	0.0232 (5)	
C5	−0.1128 (4)	0.6028 (3)	0.77204 (18)	0.0271 (5)	
H5A	−0.2300	0.5861	0.8062	0.033*	
H5B	−0.0086	0.5936	0.8202	0.033*	
C6	−0.0709 (4)	0.7759 (3)	0.7638 (2)	0.0340 (6)	
H6A	−0.1778	0.7865	0.7182	0.041*	
H6B	−0.0629	0.8560	0.8374	0.041*	
C7	0.1105 (4)	0.8203 (3)	0.7162 (2)	0.0393 (6)	
H7A	0.1270	0.9341	0.7145	0.047*	
H7B	0.0984	0.7465	0.6403	0.047*	
C8	0.2860 (5)	0.8075 (4)	0.7796 (3)	0.0547 (8)	
H8A	0.2911	0.8700	0.8567	0.082*	
H8B	0.3990	0.8524	0.7513	0.082*	
H8C	0.2806	0.6920	0.7714	0.082*	
C9	−0.0284 (3)	0.2792 (3)	0.75450 (17)	0.0208 (4)	
C10	0.1607 (3)	0.3049 (3)	0.73681 (19)	0.0262 (5)	
H10	0.1946	0.3273	0.6727	0.031*	
C11	0.2991 (3)	0.2982 (3)	0.8106 (2)	0.0305 (5)	
H11	0.4278	0.3183	0.7984	0.037*	
C12	0.2485 (4)	0.2619 (3)	0.90266 (19)	0.0305 (5)	
C13	0.0628 (4)	0.2332 (3)	0.92160 (19)	0.0304 (5)	
H13	0.0295	0.2076	0.9848	0.036*	
C14	−0.0752 (3)	0.2422 (3)	0.84734 (18)	0.0260 (5)	
H14	−0.2036	0.2226	0.8602	0.031*	

### Atomic displacement parameters (Å^2^)

**Table:**

	*U*^11^	*U*^22^	*U*^33^	*U*^12^	*U*^13^	*U*^23^
Br1	0.04362 (18)	0.0746 (2)	0.02979 (15)	0.03115 (16)	−0.00597 (11)	0.01222 (14)
N1	0.0289 (11)	0.0264 (11)	0.0266 (10)	0.0030 (9)	−0.0045 (8)	0.0088 (8)
O1	0.0634 (14)	0.0274 (11)	0.0616 (14)	0.0184 (10)	0.0132 (11)	0.0107 (9)
O2	0.0374 (11)	0.0417 (11)	0.0495 (12)	−0.0013 (9)	0.0085 (9)	0.0192 (9)
O3	0.0297 (9)	0.0443 (10)	0.0287 (9)	0.0175 (8)	0.0073 (7)	0.0199 (8)
O4	0.0251 (9)	0.0576 (12)	0.0373 (10)	0.0159 (8)	0.0081 (7)	0.0263 (9)
C1	0.0245 (11)	0.0237 (12)	0.0240 (11)	0.0047 (9)	0.0021 (9)	0.0092 (9)
C2	0.0201 (10)	0.0251 (12)	0.0200 (10)	0.0068 (9)	0.0023 (8)	0.0089 (9)
C3	0.0220 (10)	0.0262 (12)	0.0214 (10)	0.0085 (9)	0.0019 (8)	0.0104 (9)
C4	0.0272 (11)	0.0216 (12)	0.0227 (11)	0.0098 (9)	0.0024 (9)	0.0075 (9)
C5	0.0350 (13)	0.0258 (12)	0.0227 (11)	0.0098 (10)	0.0049 (10)	0.0090 (9)
C6	0.0533 (17)	0.0231 (12)	0.0267 (12)	0.0128 (11)	0.0001 (11)	0.0077 (10)
C7	0.0575 (18)	0.0270 (14)	0.0333 (14)	0.0037 (12)	0.0011 (13)	0.0140 (11)
C8	0.0475 (18)	0.055 (2)	0.060 (2)	−0.0039 (15)	−0.0035 (16)	0.0280 (17)
C9	0.0225 (10)	0.0198 (11)	0.0209 (10)	0.0072 (8)	0.0014 (8)	0.0065 (8)
C10	0.0244 (11)	0.0332 (13)	0.0236 (11)	0.0080 (10)	0.0051 (9)	0.0117 (10)
C11	0.0212 (11)	0.0392 (14)	0.0325 (13)	0.0111 (10)	0.0038 (9)	0.0112 (11)
C12	0.0318 (12)	0.0360 (14)	0.0235 (11)	0.0162 (10)	−0.0033 (9)	0.0050 (10)
C13	0.0375 (14)	0.0368 (14)	0.0222 (11)	0.0143 (11)	0.0039 (10)	0.0136 (10)
C14	0.0254 (11)	0.0322 (13)	0.0243 (11)	0.0091 (10)	0.0062 (9)	0.0124 (10)

### Geometric parameters (Å, º)

**Table:**

Br1—C12	1.894 (2)	C6—C7	1.521 (4)
N1—O1	1.213 (3)	C6—H6A	0.9900
N1—O2	1.218 (3)	C6—H6B	0.9900
N1—C1	1.492 (3)	C7—C8	1.525 (4)
O3—C4	1.270 (3)	C7—H7A	0.9900
O3—H3A	0.8400	C7—H7B	0.9900
O4—C4	1.252 (3)	C8—H8A	0.9800
C1—C2	1.528 (3)	C8—H8B	0.9800
C1—H1A	0.9900	C8—H8C	0.9800
C1—H1B	0.9900	C9—C14	1.388 (3)
C2—C9	1.512 (3)	C9—C10	1.394 (3)
C2—C3	1.545 (3)	C10—C11	1.377 (3)
C2—H2	1.0000	C10—H10	0.9500
C3—C4	1.511 (3)	C11—C12	1.383 (3)
C3—C5	1.537 (3)	C11—H11	0.9500
C3—H3	1.0000	C12—C13	1.375 (4)
C5—C6	1.528 (3)	C13—C14	1.387 (3)
C5—H5A	0.9900	C13—H13	0.9500
C5—H5B	0.9900	C14—H14	0.9500
			
O1—N1—O2	123.8 (2)	C7—C6—H6B	108.7
O1—N1—C1	118.5 (2)	C5—C6—H6B	108.7
O2—N1—C1	117.7 (2)	H6A—C6—H6B	107.6
C4—O3—H3A	109.5	C6—C7—C8	113.6 (2)
N1—C1—C2	110.98 (18)	C6—C7—H7A	108.9
N1—C1—H1A	109.4	C8—C7—H7A	108.9
C2—C1—H1A	109.4	C6—C7—H7B	108.9
N1—C1—H1B	109.4	C8—C7—H7B	108.9
C2—C1—H1B	109.4	H7A—C7—H7B	107.7
H1A—C1—H1B	108.0	C7—C8—H8A	109.5
C9—C2—C1	111.47 (17)	C7—C8—H8B	109.5
C9—C2—C3	111.60 (17)	H8A—C8—H8B	109.5
C1—C2—C3	109.94 (17)	C7—C8—H8C	109.5
C9—C2—H2	107.9	H8A—C8—H8C	109.5
C1—C2—H2	107.9	H8B—C8—H8C	109.5
C3—C2—H2	107.9	C14—C9—C10	118.4 (2)
C4—C3—C5	108.96 (17)	C14—C9—C2	120.5 (2)
C4—C3—C2	109.26 (18)	C10—C9—C2	121.08 (19)
C5—C3—C2	111.06 (17)	C11—C10—C9	121.2 (2)
C4—C3—H3	109.2	C11—C10—H10	119.4
C5—C3—H3	109.2	C9—C10—H10	119.4
C2—C3—H3	109.2	C10—C11—C12	119.2 (2)
O4—C4—O3	123.8 (2)	C10—C11—H11	120.4
O4—C4—C3	118.8 (2)	C12—C11—H11	120.4
O3—C4—C3	117.3 (2)	C13—C12—C11	121.0 (2)
C6—C5—C3	113.78 (19)	C13—C12—Br1	119.74 (19)
C6—C5—H5A	108.8	C11—C12—Br1	119.22 (19)
C3—C5—H5A	108.8	C12—C13—C14	119.3 (2)
C6—C5—H5B	108.8	C12—C13—H13	120.4
C3—C5—H5B	108.8	C14—C13—H13	120.4
H5A—C5—H5B	107.7	C13—C14—C9	120.9 (2)
C7—C6—C5	114.3 (2)	C13—C14—H14	119.5
C7—C6—H6A	108.7	C9—C14—H14	119.5
C5—C6—H6A	108.7		
			
O1—N1—C1—C2	−132.7 (2)	C5—C6—C7—C8	−58.1 (3)
O2—N1—C1—C2	48.5 (3)	C1—C2—C9—C14	−115.2 (2)
N1—C1—C2—C9	69.0 (2)	C3—C2—C9—C14	121.5 (2)
N1—C1—C2—C3	−166.69 (17)	C1—C2—C9—C10	66.1 (3)
C9—C2—C3—C4	−176.36 (17)	C3—C2—C9—C10	−57.2 (3)
C1—C2—C3—C4	59.4 (2)	C14—C9—C10—C11	−1.7 (3)
C9—C2—C3—C5	−56.1 (2)	C2—C9—C10—C11	177.0 (2)
C1—C2—C3—C5	179.63 (18)	C9—C10—C11—C12	1.4 (4)
C5—C3—C4—O4	−61.1 (3)	C10—C11—C12—C13	−0.3 (4)
C2—C3—C4—O4	60.4 (3)	C10—C11—C12—Br1	179.80 (18)
C5—C3—C4—O3	118.3 (2)	C11—C12—C13—C14	−0.5 (4)
C2—C3—C4—O3	−120.2 (2)	Br1—C12—C13—C14	179.44 (18)
C4—C3—C5—C6	−59.3 (3)	C12—C13—C14—C9	0.1 (4)
C2—C3—C5—C6	−179.7 (2)	C10—C9—C14—C13	0.9 (3)
C3—C5—C6—C7	−60.6 (3)	C2—C9—C14—C13	−177.8 (2)

### Hydrogen-bond geometry (Å, º)

**Table:**

*D*—H···*A*	*D*—H	H···*A*	*D*···*A*	*D*—H···*A*
C1—H1*B*···O1^i^	0.99	2.43	3.410 (3)	172
C1—H1*A*···O2^ii^	0.99	2.51	3.265 (3)	133
C3—H3···O3^iii^	1.00	2.69	3.679 (3)	169
C8—H8*B*···O2^iv^	0.98	2.60	3.501 (4)	153
O3—H3*A*···O4^v^	0.84	1.79	2.619 (2)	171

Symmetry codes: (i) −*x*, −*y*, −*z*+1; (ii) −*x*−1, −*y*, −*z*+1; (iii) −*x*, −*y*+1, −*z*+1; (iv) *x*+1, *y*+1, *z*; (v) −*x*−1, −*y*+1, −*z*+1.
